# What to do to beat Langerhans cell histiocytosis of bone? A narrative review and case series of radiofrequency ablation

**DOI:** 10.1530/EOR-2025-0181

**Published:** 2026-03-02

**Authors:** Mehmet Askin, Ibrahim Mehmet Goymen, Melih Oral, Mehmet Ayvaz

**Affiliations:** ^1^ Yuksekova State Hospital, Orthopedics and Traumatology Department, Hakkari, Turkey; ^2^ Hacettepe University Medical School, Orthopedics and Traumatology Department, Ankara, Turkey; ^3^ Idil State Hospital, Orthopedics and Traumatology Department, Sirnak, Turkey; ^4^ Graduate School of Biomedical Engineering, Middle East Techical University, Ankara, Turkey

**Keywords:** Langerhans cell histiocytosis, radiofrequency ablation, bone tumors

## Abstract

- Langerhans cell histiocytosis (LCH) is a myeloid neoplastic disorder in which bone is the commonly affected organ system. While treatment for its symptomatic bone lesions varies, modern minimally invasive techniques show significant advantages over traditional approaches.
- Conventional therapies present notable limitations. Curettage, while frequently used, is associated with local recurrence. The efficacy of intralesional corticosteroid injections remains uncertain, especially for lesions in the extremities or pelvis. Although effective, low-dose radiation therapy carries long-term risks and is reserved for specific cases. Systemic chemotherapy, the standard for multifocal disease, is associated with toxicity and high relapse rates.
- Radiofrequency ablation (RFA) has emerged as a superior alternative that fills this therapeutic gap. RFA is a minimally invasive procedure that uses targeted heat (60–100°C) to destroy tumor cells with curative intent. Validating prior case reports, our recent study of ten patients confirmed that RFA provides complete pain relief with no residual disease or recurrence on follow-up MRI. Importantly, no significant complications were observed in our cohort or have been reported in the literature for LCH patients treated with RFA.
- In conclusion, RFA offers a safe, rapid, and durable solution for painful LCH lesions. It should be considered a primary curative treatment for symptomatic LCH of the bone, avoiding the risks of more invasive procedures and systemic therapies.

## Clinical and pathobiological landscape of Langerhans cell histiocytosis

Langerhans cell histiocytosis (LCH), the most common histiocytosis, is a rare disorder characterized by the pathologic accumulation of dendritic cells that resemble epidermal Langerhans cells (1, 2). For decades, the fundamental nature of LCH was debated, with its prominent inflammatory infiltrate and occasional spontaneous regressions suggesting a reactive or immune-dysregulatory process (1). This ambiguity was reflected in the historical term ‘histiocytosis X’ (3).

However, a paradigm shift has occurred, recasting LCH as a clonal myeloid neoplastic disorder (4). This modern conception is driven by the discovery of recurrent, activating somatic mutations in the mitogen-activated protein kinase (MAPK) signaling pathway (5). The landmark discovery was the BRAF V600E mutation, present in the pathogenic cells of over 50% of LCH patients (6). These mutations cause the MAPK pathway to be continuously active, leading to uncontrolled cell growth and proliferation (5). This genomic evidence firmly reclassifies LCH as a myeloid neoplasm. It provides a compelling rationale for applying established oncologic principles of local tumor eradication, such as radiofrequency ablation (RFA), with curative intent for localized disease (4). Today, some debate exists about the position among diseases: is it one of the most common pediatric cancers, or is it just another rare disorder (7)?

Granulomatous lesions formed by CD207-positive histiocytes in Langerhans cell histiocytosis (LCH) can affect nearly every organ in the body (8). Bones, lungs, and skin are among the most frequently involved organs.

LCH can affect individuals at any age; its epidemiological features vary significantly between children and adults. According to international expert consensus guidelines, the estimated annual incidence of LCH in children is approximately 4–5 cases per million, while in adults, it is markedly lower, around 1–2 cases per million (9, 10). The disease shows a slight male predominance in pediatric populations, but this gender bias is less consistent in adult-onset LCH. Notably, adult patients are often underdiagnosed due to the heterogeneous clinical presentation and lower awareness among clinicians. Smoking has been strongly associated with pulmonary LCH in adults, suggesting that environmental factors may influence disease expression in this group. These variations underscore the need for age-specific diagnostic and management strategies in LCH.

### Clinical spectrum and diagnosis of LCH

The clinical presentation of LCH is highly variable, ranging from a single, self-resolving lesion to aggressive, life-threatening multisystem disease (2). It shows itself as mainly unifocal, but a disseminated form is primarily observed in infants (11). The disease is broadly categorized based on the number of organ systems involved (2):

- **Single-system LCH:** disease is confined to one organ or system. This can be unifocal (a single lesion) or multifocal (multiple lesions in one system, e.g., multiple bones).
- **Multisystem LCH:** the disease involves two or more organ systems. This form is further stratified by the involvement of ‘risk organs’ – the liver, spleen, and hematopoietic system – which is associated with a higher risk of mortality (2, 4).

Historically, different presentations were known by eponyms: eosinophilic granuloma for isolated bone lesions, Hand–Schüller–Christian disease for a chronic, multifocal form (classically a triad of skull lesions, exophthalmos, and diabetes insipidus), and Letterer–Siwe disease for the severe, disseminated form in infants (1, 3).

Bone is the most frequently affected organ system, involved in approximately 30–80% of cases (2, 12). The characteristic radiographic appearance is a ‘punched-out’ osteolytic lesion, which can mimic osteomyelitis, Ewing sarcoma, or other tumors, making biopsy essential for diagnosis (1). Specific signs include vertebra plana (vertebral body collapse), a ‘hole-within-a-hole’ appearance in flat bones, and an aggressive, permeative look in long bones (3).

A definitive diagnosis depends on histopathology from a tissue biopsy (2). The gold standard is the identification of characteristic Langerhans cells combined with positive immunohistochemical (IHC) staining for CD1a and/or CD207 (Langerin) (1, 2). Even when clinical and imaging findings are highly suggestive of LCH, a biopsy of the lesional tissue is recommended to confirm the diagnosis and determine the mutational status of BRAF or other MAPK–ERK pathway genes (10). Once diagnosed, a comprehensive staging workup, preferably including FDG-PET/CT, is essential to distinguish unifocal disease from multisystem disease that requires systemic treatment (4).

## Management of skeletal LCH

The extent of the disease dictates the management of LCH bone lesions, the location of the lesion(s), and the severity of symptoms (2). A multidisciplinary approach involving oncologists, orthopedic surgeons, and interventional radiologists is strongly recommended (1).

### Unifocal and multifocal bone disease

Unifocal bone lesions are the most common form of LCH and may have a good prognosis (2). Many of these lesions might be self-limiting and may resolve spontaneously over months to years, with healing sometimes initiated by the diagnostic biopsy itself (1, 3).

#### Indications for treatment

For asymptomatic lesions, observation is a reasonable approach (1). Indications for active treatment include significant pain, restriction of mobility, the presence of a lesion in a weight-bearing bone, an impending pathologic fracture, or neurologic compression (2, 3).

#### Treatment options

A significant therapeutic gap exists for patients with symptomatic bone lesions who require more than simple observation but may wish to avoid more invasive procedures, such as open surgery, or the long-term risks of radiation therapy. Minimally invasive techniques are therefore highly attractive.

- **Biopsy and curettage:** in many accessible lesions, simple curettage performed during the initial diagnostic biopsy is sufficient to induce healing (2). However, certain locations present significant challenges. Some lesions may be difficult to reach (such as pelvic lesions), while periarticular lesions may pose a higher iatrogenic damage to important structures during an open approach.
- **Intralesional corticosteroid injection:** the injection of methylprednisolone directly into the lesion is a well-described, minimally invasive option that can promote healing and provide pain relief (2, 3).
- **Low-dose radiation therapy:** radiation can be effective, with local control rates over 90% (13). However, due to long-term risks, it is generally reserved for symptomatic, inoperable lesions in functionally critical locations (1, 3).
- **Chemotherapy:** the discovery of BRAF and MAP2K1 mutations in LCH has led to targeted therapies acting upon the RAS/RAF/MEK/ERK pathway. Small series and anecdotal case reports of refractory and relapsed LCH have shown responses to the BRAF inhibitors, vemurafenib and dabrafenib (14, 15). Myelotoxicity of BRAF inhibition seems to be less than that of nucleoside analogs (cladribine and clofarabine) or hematopoietic stem cell transplantation (16).
- **Radiofrequency ablation (RFA):** RFA has emerged as a safe, effective, and minimally invasive alternative for treating painful LCH lesions (17, 18). It offers the benefits of rapid pain relief, a short recovery time, and a curative outcome in appropriate candidates (19). Our recent work further solidifies the role of RFA as a definitive curative therapy for both single and multiple bone LCH lesions.
- **Curettage, grafting, and internal fixation:** in patients with pathological fractures or impending fractures due to the lesion, curettage, grafting, and internal fixation is the treatment of choice (20).

### ‘Special site’ and systemic disease

Systemic therapy is indicated for multifocal bone disease and lesions involving ‘CNS-risk’ sites (e.g., skull base, orbital bones, and temporal bones) to mitigate the risk of long-term sequelae such as diabetes insipidus and neurodegeneration (2, 4). The standard regimen is typically vinblastine and prednisone (2).

## Radiofrequency ablation: could it be a definitive curative therapy for bone lesions?

Percutaneous thermal ablation is a cornerstone of modern interventional oncology, and RFA is the most established of these techniques for bone lesions (21). It offers a minimally invasive means to destroy tumors using thermal energy with curative intent (21).

### Principles and technology of RFA

RFA employs a high-frequency alternating current passed through a needle electrode placed into the tumor (17). The tissue’s resistance to the current generates intense frictional heat, raising the local temperature to between 60 and 100°C, causing irreversible cell death (18). The procedure is performed under imaging guidance, typically CT, to ensure precise and safe placement of the electrode (17).

Recent technological advancements, such as navigational bipolar RFA systems, have significantly enhanced safety and efficacy (19). These systems allow for precise steering of the electrode tip and real-time temperature monitoring, which is crucial for treating irregularly shaped tumors or those near critical structures, such as nerves (22).

### Clinical evidence for RFA in LCH: from case reports to cohort-level validation

The evidence supporting RFA for LCH has evolved from promising case reports to more robust clinical data, establishing it as a powerful therapeutic modality.

### Foundational case evidence

Initial case reports provided the foundational proof-of-concept. Corby *et al.* (17) were among the first ones describing rapid pain relief and radiographic healing in two patients after RFA therapy. This was followed by a detailed report from Papavasiliou *et al.* (18) on a 16-year-old male with a femoral lesion, demonstrating not only immediate pain relief but also complete and durable radiological resolution with no recurrence at a 48-month imaging follow-up. Concurrently, Tomasian *et al.* (19) reported the successful and safe curative treatment of a painful lesion in the challenging supra-acetabular region using a navigational bipolar RFA system, highlighting the importance of advanced technology.

While these reports were encouraging, they were limited to a small number of cases. Our recent retrospective single-center study significantly strengthens this evidence base.

### Our study: methods and study design

**Figure 1:**
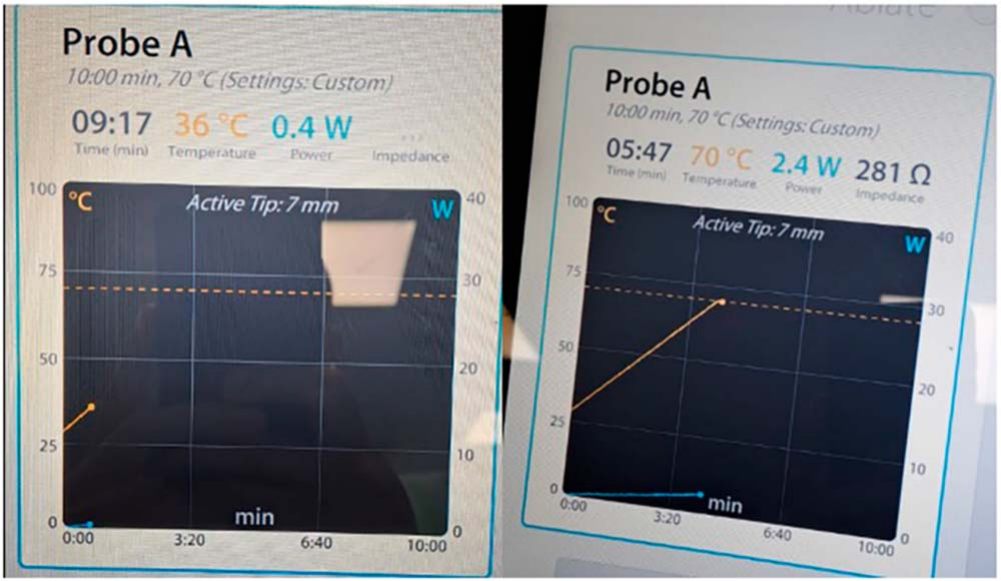
Radiofrequency ablation device screen. The temperature at the tip of the probe can be monitored in real time via the device’s control panel. After the target temperature is set, the desired temperature (90–95°C) is reached in approximately 4.5 min, and radiofrequency ablation is subsequently applied within the lesion for a duration of 6 min.

**Figure 2:**
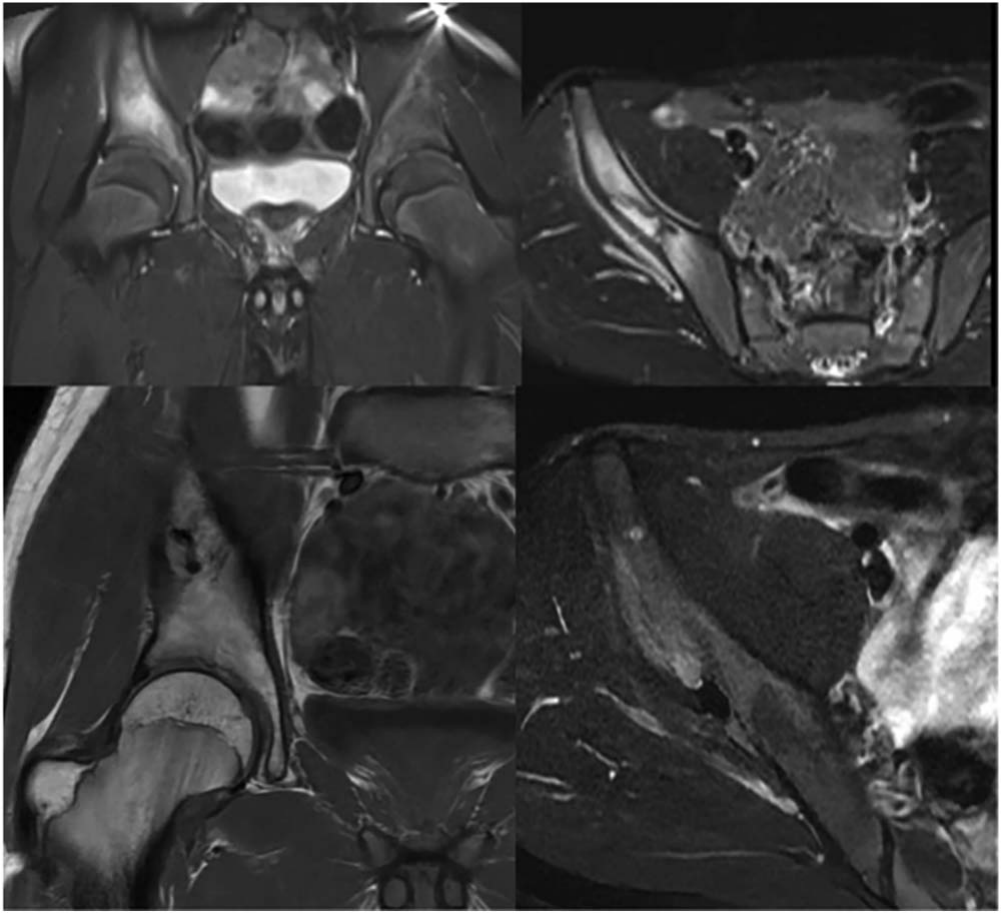
Preoperative and postoperative MRI images of a patient diagnosed with LCH in the iliac bone.

**Figure 3:**
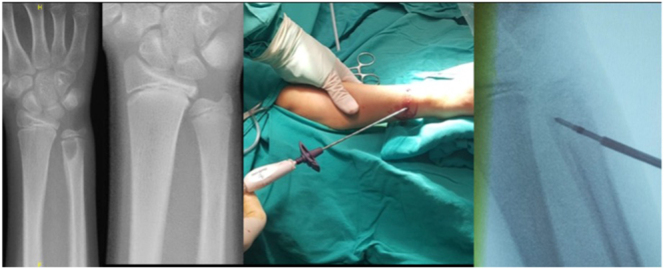
Radiographs of an 11-year-old female patient diagnosed with LCH before and after RFA and application of RFA to the lesion with intraoperative fluoroscopy.

**Figure 4:**
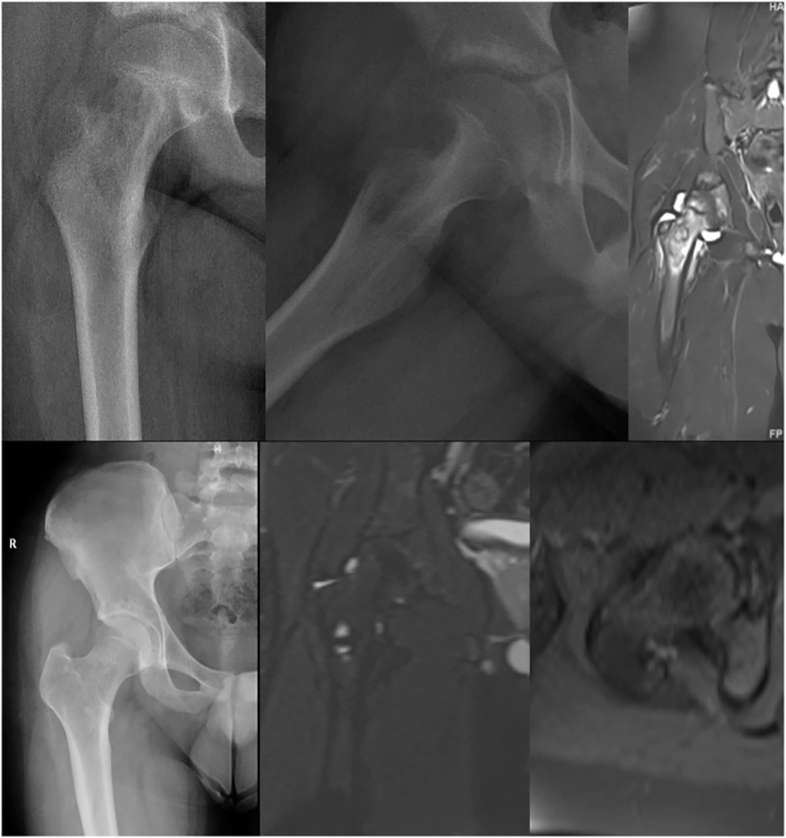
Radiographs and MRI images of a 19-year-old male patient diagnosed with LCH in the right proximal femur before RFA and after a 4-year follow-up.

This retrospective single-center study included 20 patients diagnosed with LCH, confirmed pathologically and followed up for at least one year. Ethical approval was obtained from the ethics committee of the Hacettepe University Medical School (SBA 24/035). Patients presenting with pain symptoms in various bone regions were monitored for pain severity using the numerical rating scale (NRS) during pre-procedure and follow-up evaluations. Following initial presentations, plain radiographs and MRI were performed, and closed bone biopsies were taken to confirm the pathological diagnoses of the patients. Patients with multiple lesions were referred to an oncologist and received appropriate chemotherapy treatment. Subsequently, ten patients underwent RFA therapy targeting the lesions in the affected regions, performed at 90–95°C for six minutes using the Medtronic OsteoCool™ bipolar radiofrequency ablation system. This system is shown in Fig. 1. The treatment protocol is similar to radiofrequency ablation treatments previously applied in tumor patients (23) (Fig. 1). These ten patients also underwent follow-up MRI, and their pain levels were reassessed. The largest dimensions of the lesions were measured on coronal, sagittal, and axial planes during the diagnostic MRI, with a similar protocol followed during subsequent evaluations. Preoperative and follow-up direct radiographs and magnetic resonance imaging of some patients in our study are shown in Figs 2, 3, and 4.

This study aims to evaluate the power of RFA as a curative therapy for single or multiple lesions caused by LCH involving different parts of the body. Since LCH is a rare disease, the current literature lacks evidence regarding the efficacy of the technique. Furthermore, by using follow-up imaging modalities (MRI), this study has evaluated the presence of residual lesions and thus the risk of recurring disease after the RFA procedure.

### Our study: results

In this study, ten patients who underwent RFA for LCH presented with skeletal lesions located in various anatomical regions. At the time of diagnosis, all patients experienced moderate to severe pain, as quantified by the NRS, underscoring the clinical burden of osseous LCH. Post-procedural follow-up using magnetic resonance imaging (MRI) demonstrated no evidence of residual disease in any of the cases, suggesting complete local disease control. Clinically, all patients reported substantial pain relief, with pain levels decreasing to a minimal level or absence during subsequent evaluations. Importantly, there were no instances of significant complications, including infection, neurovascular injury, or procedure-related morbidity.

These findings underscore the therapeutic potential of RFA not merely as a palliative intervention but as a definitive and curative treatment modality for solitary and multifocal skeletal LCH lesions. By demonstrating radiological remission and symptomatic improvement across all cases, this study contributes important clinical evidence to a relatively underexplored area in the current literature and supports the integration of RFA into the therapeutic armamentarium for osseous LCH.

The key details from published cases, including our findings, are summarized in Tables 1 and 2.

**Table 1:** Details of patient data in our study.

PT#	Age	Sex	FU (mo)	Lesion localization	RFA	CT	RT	MRI lesion size (mm)		Complications
								Preop	FU	
1	74	F	60	Femur and cranium	No	Yes	No	51 × 50	31 × 24	No
2	57	F	36	Tibia and cranium	No	Yes	No	103 × 75	55 × 40	No
3	35	M	72	Femur and L2 body	No	Yes	10	24 × 21	13 × 11	No
4	6	M	36	Ilium, humerus, and cranium	No	Yes	No	45 × 40	71 × 66	No
5	18	M	108	Tibia and cranium	No	Yes	2	20 × 19	16 × 17	No
6	20	M	72	Femur	No	Yes	No	31 × 25	15 × 10	No
7	44	F	48	Humerus	No	Yes	No	32 × 23	25 × 24	No
8	11	F	84	Scapula and femur	No	Yes	No	11 × 6	0	No
9	6	M	56	Femur	No	Yes	No	90 × 40	0	No
10	11	F	12	Sternum, acetabulum, and humerus	No	Yes	No	15 × 10	0	No
11	19	M	96	Femur	Yes	No	No	23 × 14	0	No
12	11	F	36	Ulna	Yes	No	No	11 × 8	0	No
13	5	F	48	L2 body	Yes	No	No	24 × 20	0	No
14	37	M	72	Sacrum and cranium	Yes	No	13	68 × 16	0	No
15	15	M	12	Clavicle	Yes	No	No	11 × 7	0	No
16	11	M	12	Ilium and cranium	Yes	No	No	26 × 17	0	No
17	43	F	96	T8 body	Yes	No	No	16 × 7	0	No
18	14	M	12	İlium	Yes	No	No	38 × 17	0	No
19	8	M	14	Cranium, T6 body, and clavicle	Yes	No	1	18 × 10	0	No
20	11	F	15	Femur	Yes	Yes	No	37 × 18	0	No

PT#, patient number; FU, follow-up; mo, months; RFA, radiofrequency ablation; CT, chemotherapy; RT, radiotherapy; and MRI, magnetic resonance imaging.

**Table 2:** Detailed comparative analysis of RFA cases in LCH of bone.

Study	Patient cohort	Lesion details	RFA system/technology	Procedural details	Follow-up	Outcome
Our study	9 patients	Single and multiple painful lesions in various skeletal locations	Standard RFA	CT-guided; heated to 90–95°C for 6 min	At least 1 year (clinical and MRI)	Complete pain relief. No residual disease on follow-up MRI. No recurrence
Papavasiliou *et al.* (18)	16-year-old male	Painful 13 × 24 mm lytic lesion; right femoral diaphysis	AMICA RF (11-gauge electrode)	CT-guided; heated to 90–94°C for 8 min	48 months (imaging); 6 years (phone)	Immediate and complete pain relief. Complete radiographic healing. No recurrence
Tomasian *et al.* (19)	10-year-old male	Painful unifocal lesion; supra-acetabular iliac bone	Navigational bipolar RFA system	Percutaneous, navigational ablation	Not specified	Technical success, safe, and achieved cure
Corby *et al.* (17)	2 patients	Painful lesions in ischium and femoral neck	StarBurst XL (unipolar multi-tined)	CT-guided; heated to 90°C for 3–5 min (x2 cycles)	12 and 18 months	Rapid pain relief. Radiographic healing

## Discussion

The understanding of LCH has evolved from a mysterious inflammatory condition to a defined myeloid neoplasm. This has solidified the rationale for using local, curative-intent therapies for isolated and multifocal bone disease. For symptomatic LCH of bones, a spectrum of treatments exists, from simple curettage and steroid injections to systemic chemotherapy for high-risk locations.

Curettage is one of the most common treatment methods for solitary lesions. Although some case series and research recommend the procedure especially for accessible lesions, such as extremities, jaw, or skull, other studies show that local recurrence is a common complication, which results in the need for further treatment (24, 25, 26, 27, 28). Due to the risk of local recurrence observed in patients treated with curettage alone, radiofrequency ablation following biopsy during primary surgery may be considered a curative and cost-effective treatment option. Furthermore, curettage for spinal LCH is less favored due to higher complication rates and worse outcomes compared to conservative management or posterior instrumentation without curettage (29, 30).

Intralesional corticosteroid administration is a frequently employed treatment modality, particularly for localized Langerhans cell histiocytosis (LCH) lesions. However, the efficacy and safety of this approach have predominantly been evaluated in case series focusing on lesions of the mandible and cranial bones (31, 32). Its reliability remains unclear. The limited follow-up periods in existing studies, inconsistencies regarding optimal steroid dosage, and variable post-treatment radiological changes reported in some investigations contribute to ongoing uncertainty about the overall efficacy of this treatment (32, 33).

Chemotherapy is a widely used treatment option, generally reserved for systemic diseases or patients with multiple lesions. Some widely accepted drug regimens, even though they lead to high rates of lesion resolution and disease control, could cause toxicity or at least have lower effectiveness than could be accepted. For example, the vinblastine/prednisone therapy is associated with low effectiveness and toxicity in adults (34). On the other hand, a relatively new study indicates that intensifying chemotherapy (e.g., higher doses of vincristine) may not improve outcomes and may increase side effects (35). Moreover, many studies suggest that, if the lesions could be treated with more local approaches, systemic modalities may not be needed, considering the potential profit/harm ratio (36, 37, 38). A study published in 2022 with 20 patients including both unifocal and multifocal diseases suggests that chemotherapy is a viable option for the treatment of the bone lesions (39). On the other hand, the same paper shows that either many complications, including nerve damage, or chronic pain was observed or regimens had to be changed due to the ineffective results.

Radiotherapy is another treatment modality of bone lesions aiming to achieve effective therapy for bone lesions. Typical total doses range between 8 and 24 Gy delivered in parts (40). In addition, some studies show that there are recurrences up to 20% in the following years (40). Still, about 40% of the patients suffer from symptoms such as pain after the radiotherapy (40). On top of that, there is always a tendency to choose more ‘local and secure’ treatment modalities among patients.

RFA has emerged as a superior minimally invasive treatment. While early case reports were promising, they lacked the weight of larger series. Our study provides this crucial validation. The demonstration of complete pain resolution and, most importantly, the absence of residual disease on follow-up MRI in all ten treated patients provide strong evidence for RFA as a definitive curative therapy. The excellent safety profile, with no major complications, further strengthens its position as a first-line option for appropriate candidates. One of the main concerns regarding radiofrequency ablation treatment is the potential risk of physeal injury in pediatric patients with lesions located close to the physis. There are studies demonstrating that, in lesions located close to the physis, radiofrequency ablation is a safer and more effective treatment modality compared with curettage (23, 41). When the application protocol is strictly followed, radiofrequency ablation has been reported not to adversely affect the growth potential of the physis.

A review of previous studies reveals that mostly case reports and a few case series have been published involving patients with LCH treated with RFA.

In the case report by Papavasiliou *et al.* (18), a 16-year-old male patient underwent RFA of a femoral diaphyseal lesion under CT guidance, using a temperature of 90–94°C for 8 min. Follow-up over two years revealed no recurrence, and the patient experienced significant pain relief. Similarly, in our study, no recurrence was observed in any of the ten patients during a minimum follow-up period of one year.

In the case report published by Tomasian *et al.* (19), the safety of bipolar RFA applied to the supra-acetabular region was emphasized. Consistent with these findings, our study also demonstrated the safe completion of RFA procedures under CT guidance in ten patients with lesions at various anatomical sites, without any complications.

In the case series published by Corby *et al.* (17), RFA was performed in two patients with painful lesions located in the ischium and femoral neck, using two cycles of 3–5 min at an average temperature of 90°C. In our study, a single cycle of RFA was applied at a temperature of 90–95°C for 6 min. Based on the pain scores and recurrence rates observed in our cohort of ten patients, this protocol appears to be effective and clinically successful.

In general, RFA is considered a low-risk procedure. However, several potential complications merit attention. Pain during or after the procedure has been reported by a significant minority of patients, with rates reaching up to 21% (42). In RFA procedures applied to visceral organs, self-limiting hematoma is among the most common complications (43). However, no complications have been reported in the literature among LCH patients treated with RFA, nor were any observed in our own patient cohort.

While RFA has been employed in the treatment of various benign bone tumors (44), we did not identify any prior studies specifically investigating the efficacy of RFA in patients with LCH. In our series of ten patients treated with RFA, no complications or recurrences were observed, and pain scores were found to be significantly reduced. These findings suggest that RFA may represent an effective treatment modality for patients with LCH.

## Conclusion

RFA effectively fills a critical gap between simple observation and more invasive surgical procedures or radiation. It offers patients a safe, rapid, and durable solution to painful LCH lesions, allowing for a quick return to normal activities with minimal morbidity. Based on the cumulative evidence, now bolstered by our cohort study, RFA should be considered a primary curative treatment modality for patients with symptomatic single or multiple LCH bone lesions. Future prospective, multi-center studies are warranted to further cement its role in the standard of care.

## ICMJE Statement of Interest

The authors declare that there is no conflict of interest that could be perceived as prejudicing the impartiality of the work reported.

## Funding Statement

This research did not receive any specific grant from funding agencies in the public, commercial, or not-for-profit sectors.

## Data availability

The data used and/or analyzed during the current study are available from the corresponding author on reasonable request.
